# Understanding indications and defining guidelines for breast magnetic resonance imaging

**DOI:** 10.4102/sajr.v22i2.1353

**Published:** 2018-10-30

**Authors:** Peter K. Schoub

**Affiliations:** 1 Department of Radiology, Parklane Radiology, Johannesburg, South Africa

## Abstract

Magnetic resonance imaging (MRI) of the breast is the most sensitive imaging modality for detecting cancer. With improved scan resolution and correctly applied clinical indications, the specificity of breast MRI has markedly improved in recent years. Current literature indicates an overall sensitivity for breast MRI of 98% – 100% and specificity of 88%. By comparison, the sensitivity and specificity for mammography is in the region of 71% and 98%, respectively. In particular, the very high negative predictive value (NPV) of breast MRI, which approaches 100%, is hugely useful in establishing absence of disease. Furthermore, the ability to accurately delineate viable cancer by way of combining both morphological and functional (contrast enhancement) capabilities means that MRI is the best tool we have in terms of local cancer staging and identifying residual or recurrent disease. The high NPV also means that breast MRI is uniquely capable of ruling out cancer or high-grade ductal carcinoma in situ in appropriate circumstances. I hope that the following guidelines that are based on those of the American College of Radiology and the European Society of Breast Imaging in addition to multiple review articles will provide some assistance to radiologists in terms of the correct indications for breast MRI. There are few formal guidelines in South Africa for the usage of breast MRI. In fact, there is a general paucity of guidelines in the international radiology world. The role of breast MRI in high-risk screening and identification of the primary in occult breast cancer is universally accepted. Thereafter, there is little consensus. By using some general guidelines, and bringing MRI into the discussion of multidisciplinary breast cancer management, good clinical practice and consistent decision-making can be established.

## Introduction

Breast magnetic resonance imaging (MRI) has been part of the breast imaging armamentarium for at least 20 years. It was realised early on that MRI with the use of intravenous gadolinium contrast was a highly sensitive tool for differentiating cancer from background tissue. As opposed to relying on morphologic changes as seen with mammography, contrast-enhanced MRI is effective because it relies on cancer-associated changes at the functional level, most particularly the neovascularity and abnormal capillary permeability that accompany malignancy.^1^

With a sensitivity between 98 and 100%, and a specificity of up to 88%, MRI is a far more accurate modality in diagnosis and characterization of breast malignancy than either mammography or breast ultrasound^1,2,3^. The negative predictive value (NPV) of MRI is close to 100% and probably its most powerful attribute, as it provides the ability to unequivocally exclude malignancy^4,5,6^. The following review article uses guidelines from the American College of Radiology (ACR)^7^ and the European Society of Breast Imaging (Eusobi)^8,9^ as a foundation for setting out breast MRI indications. This review includes data and opinion from articles written by the most experienced breast MRI experts around the world in order for readers to understand the benefits and limitations of MRI scanning of the breast. Adherence to formal guidance and rational protocols in addition to collaboration amongst the specialists comprising a breast cancer multi-disciplinary team, will ensure appropriate implementation of a breast MRI program^10,11^.

Many of the changes associated with cancer on mammography relate to hypoxia and regression – desmoplastic reaction, spiculation and micro-calcifications. This means that many of the most typical breast cancers found on mammogram are the least biologically active.^12^ The higher grade and more aggressive subtypes, for example, triple negative cancers, may be less conspicuous or at least appear similar to benign entities.^13^

Magnetic resonance imaging, on the other hand, demonstrates best the most biologically active cancers (invasive and intra-ductal) and with addition of kinetic enhancement assessment, the ability to differentiate benign-appearing cancers from true benign lesions is further improved.^5,12^ Kinetic or dynamic enhancement refers to the progressive enhancement of a mass or non-mass lesion. It is plotted as the time: intensity curve on a graph. Modern software calculates an average for the entire enhancing area. The intensity (percentage) of initial enhancement in the first minute and the degree of contrast persistence or washout are reflected in the curve. The more intense (rapid) the initial enhancement and the more rapid the washout, the higher the likelihood of malignancy. For ease of use, a colour map overlay representing the type of dynamic enhancement is displayed over the area of interest. It is very important to realise that kinetic enhancement assessment is not always accurate and there is a considerable overlap between benign and malignant entities. It should not be used to downstage lesions but can be helpful in upstaging them. It is a valuable tool when used in conjunction with morphology (shape, outline) to determine the likelihood of malignancy.^1,14^

Although breast MRI was recognised early on to be highly sensitive, there was a lack of specificity. A combination of high sensitivity and suboptimal specificity resulted in too many false positives. Consequently, MRI was largely written off as an accurate and feasible investigation other than in certain specific instances.

Recent advances include dedicated multi-channel breast coils, better fat suppression, higher resolution scans and computer-aided detection (CAD) programmes that allow better use of kinetic assessment.^14,15^ In addition, evidence-based descriptors in the last two editions of the Breast Imaging, Reporting and Data System (BI-RADS) manual have standardised breast MRI assessment and reporting.^16^ The BI-RADS atlas which is produced by the American College of Radiology (ACR) describes various imaging features on each modality that indicate higher or lower suspicion of a cancer. Breast MRI has been included in the last two editions of the BI-RADS atlas.

These enhancements have meant better specificity and the ability to expand the role of breast MRI.

Furthermore, the ability to accurately biopsy lesions^17^ and insert localisation wires under MRI guidance has dramatically improved the value of pre-treatment staging MRI.^12^

Magnetic resonance imaging monitoring of neoadjuvant chemotherapy (NAC) in appropriate situations^18^ is also being increasingly adopted and will be further discussed below.

This article focuses on the best evidence we have. Breast MRI is still evolving and long-term studies of survival outcomes are limited. Many large studies are being conducted and much work is being directed at making it even more applicable, not to mention affordable. This includes abbreviated contrast-enhanced sequences and non-contrast scans utilising diffusion-weighted imaging (DWI).^15^

Fully understanding the strengths and limitations of MRI, allows us to better define indications for the use of MRI to detect, locally stage, and monitor treatment of breast cancer (see Table 1).

**TABLE 1:** Indications for breast magnetic resonance imaging.

Strength of indication	Indication type
Absolute indications	High-risk screening
	Occult breast cancer
Relative indications	Equivocal results on mammogram and ultrasound – problem solving
	Pre-operative staging
	Post-operative and/or post-treatment
	Implant assessment
	Treatment (neoadjuvant) monitoring
	Dense tissue

Note: Based primarily on ACR appropriateness criteria^7,18^ and The European Society of Breast Imaging (EUSOBI) Breast magnetic resonance imaging Guidelines,^8,9^ as well as all attached references.

## High-risk screening

All patients at high risk of developing breast cancer during their lifetime should undergo MRI imaging in addition to mammogram and ultrasound.^19^ Magnetic resonance imaging has been shown to be far more sensitive than the other breast imaging modalities. The excellent sensitivity and high negative predictive value (NPV) makes MRI the ideal screening test in this population. High-risk patients also tend to get breast cancer at a younger age, have denser breast tissue and are more likely to get high-grade cancers.^20,21,22^

**FIGURE 1:**
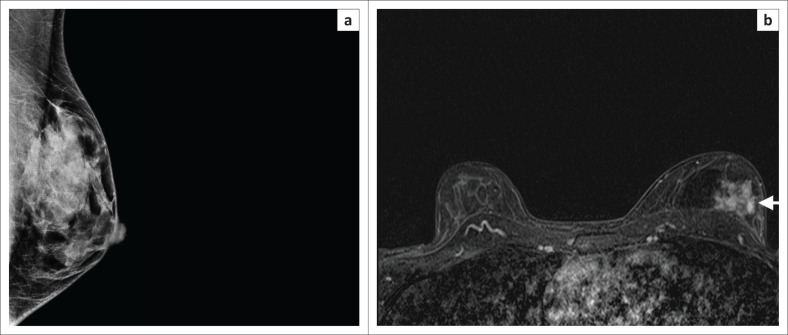
(a) The left medio-lateral oblique view mammogram of a 38-year-old woman who is a breast cancer susceptibility gene mutation carrier showing heterogeneously dense tissue but no discernible signs of cancer. (b) Magnetic resonance imaging in the same patient shows a 4 cm area of non-mass enhancement in the posterior left breast (arrow). Histology: High-Grade Ductal Carcinoma in Situ (DCIS).

Mammography is generally not offered to women younger than 35 on account of radiation exposure concerns and the higher density of tissue in younger women. The sensitivity of mammography in dense tissue can be as low as 40%.^3^ Magnetic resonance imaging, on the other hand, involves no radiation and is largely unimpeded by dense tissue (see Figure 1).

The following risk factors are absolute indications screening breast MRI^23^:

- breast cancer susceptibility gene (BRCA) mutations (including being a first-degree relative of a person with known BRCA mutation)
- other genetic disorders
- chest radiation for lymphoma
- >20% lifetime risk.

The following factors contribute towards the risk of developing breast cancer^24^:

- Family:
  ■ First-degree relatives are most important although some models also look at other relatives.
  ■ Number of relatives and age at diagnosis.
  ■ Ashkenazi or Afrikaans heritage.^25,26^
- previous biopsy where a high-risk lesion was found, for example, atypical ductal hyperplasia (ADH) and lobular carcinoma in situ (LCIS)
- nulliparity
- dense breast tissue
- early menarche or late menopause
- hormone replacement therapy
- obesity^27^
- personal history of breast cancer.^28^

### Calculators

Online calculators using a variety of different models allow users to calculate annual and lifetime risk of developing breast cancer. Most recommendations for high-risk screening, using MRI, suggest a threshold of 20% or more lifetime risk. The different models take into account various risk factors to establish likely risk. The two most frequently used calculators are:

- the IBIS calculator based on the Tyrer–Cuzick model (http://ibis.ikonopedia.com/)
- The Breast Cancer Risk Assessment Tool (BRISK) based on the Gail model (https://www.cancer.gov/bcrisktool/).

## Occult breast cancers

**FIGURE 2:**
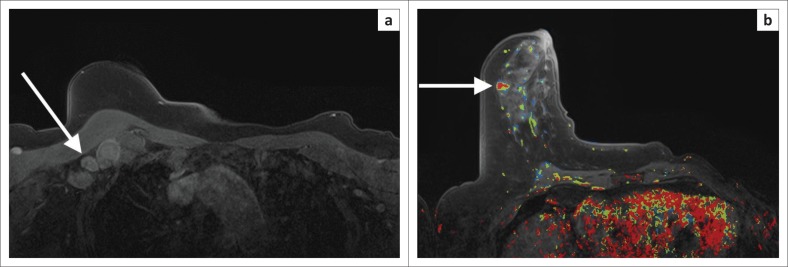
Mammogram and breast ultrasound (not shown) did not reveal any pathology in either breast. Clinically, there were enlarged lymph nodes in right axilla and lymph node biopsy revealed metastatic adenocarcinoma. (a) Axial dynamic post-contrast MRI shows multiple enlarged, enhancing lymph nodes (white arrow). (b) A small, irregular enhancing mass is evident in the anterior half of the right breast. Dynamic post-contrast scan with kinetic colour overlay demonstrating mostly red (i.e. washout that is highly suspicious for malignancy). On kinetic overlay maps, red reflects washout, yellow plateau and blue persistent features on delayed post-contrast scans. Magnetic-resonance-imaging-guided biopsy was performed. High-grade invasive ductal carcinoma confirmed on histology.

Occult breast cancers are those that are not identified clinically, on mammogram or ultrasound even though there is evidence of breast malignancy by way of metastatic lymph node disease.^29,30,31^ (see Figure 2). Bloody nipple discharge without clinical or radiological evidence of underlying breast cancer may also be considered as a feature of an occult breast cancer.^32,33^ Magnetic resonance imaging sensitivity for identifying occult breast cancer is in the range of 83% – 86%.^31^

## Pre-operative staging

**FIGURE 3:**
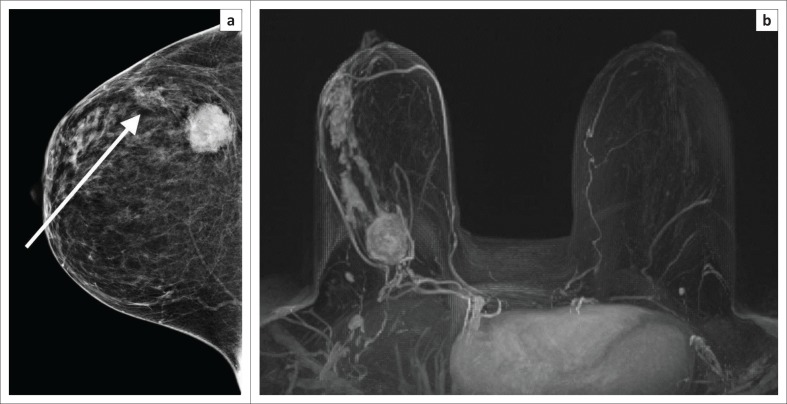
(a) Mammogram of a 56-year-old woman with a large cancer in the posterior outer aspect of the right breast. There is asymmetry and subtle distortion anterior to the mass (white arrow). This is suspicious for intra-ductal extension (associated ductal carcinoma in situ). The extent on mammogram measures 6 cm. (b) Post-contrast magnetic resonance imaging axial maximal intensity projection (MIP) shows extensive segmental non-mass enhancement extending all the way from the posteriorly situated mass to the retroareolar space. Extent is 11 cm.

**FIGURE 4:**
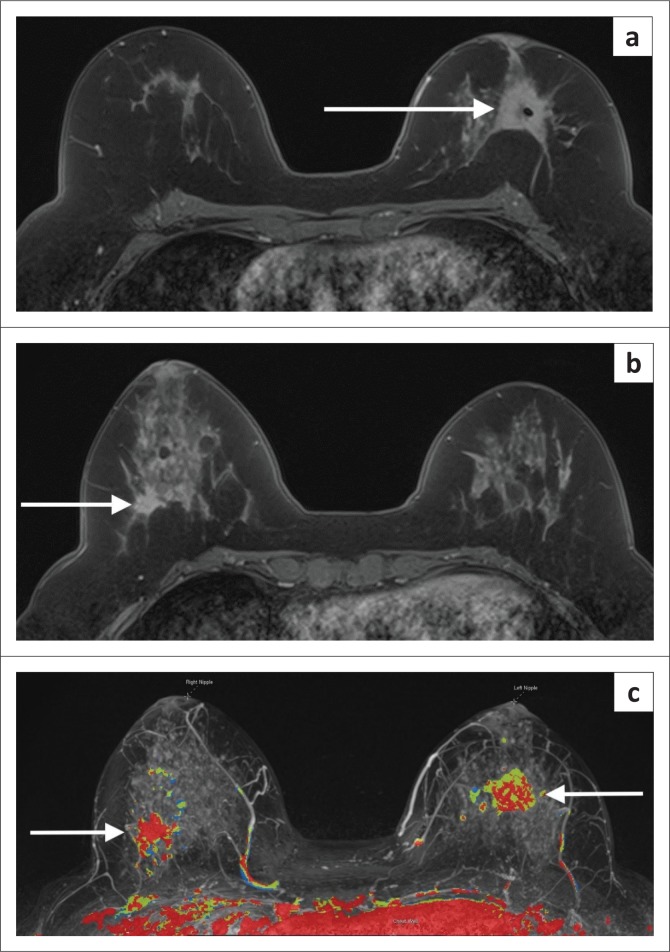
Post-contrast magnetic resonance imaging in a 40-year-old BRCA mutation carrier. (a) Large spiculated, enhancing mass in left central breast. (b) Irregular, spiculated mass in right outer breast. The mass in the right breast was occult on mammogram. (c), MIP with kinetic colour overlay showing both masses with malignant enhancement features (white arrows). Both masses were confirmed histologically as high-grade invasive carcinomas. BRCA, BReast CAncer susceptibility gene.

Magnetic resonance imaging in a pre-operative or pre-treatment role posits the tempting notion of ‘more information translates into better treatment outcomes. MRI has been shown to identify additional disease (not evident on mammogram or ultrasound) in the same breast as the primary cancer in 15–27% of cases and additional disease in the contralateral breast in 3–6% of cases^4^ (see Figures 3 and 4). However, the literature so far is ambiguous and the topic remains controversial.^34^ The two predominant end points considered are reoperation rates and disease-free survival.^12^ Several earlier studies, the comparative effectiveness of MRI in breast cancer (COMICE)^35^ and the pre-operative MRI for early-stage breast cancer (MONET)^36^ trials, showed no improvement in either. More recent literature^37,38^ confirms better reoperation rates in patients who had pre-operative MRI, which is better at tumour demarcation, identification of satellite lesions and intra-ductal extension, resulting in fewer positive margins post primary surgery.

The Multicentre International Prospective Meta-Analysis (MIPA)^39^ trial is evaluating the role of pre-operative MRI and is showing positive results in terms of surgical benefit with minimal increase in mastectomy rate.

It is important to realise that certain cancer subgroups benefit more from pre-treatment MRI. The general principle is that higher grade, worse prognosis (histological type and molecular subtype) cancers in younger patients with dense tissue, are most likely to benefit from pre-operative MRI (see Table 2).

**TABLE 2:** Appropriateness for pre-operative magnetic resonance imaging.

Inappropriate MRI	Appropriate MRI
Clearly for mastectomy (may still be needed to rule out nipple invasion)	For BCT
ER/PR +ve	Triple negative/HER-2/luminal B
Fatty breasts	DCIS (HG)
-	Invasive lobular
-	Dense breasts
-	Young patients

*Source*: Kuhl et al.^37^; Sardanelli^38^; MIPA^39^; Ha et al.^40^; Derias et al.^41^; Bae et al.^42^; Grimm et al.^43^; Eun et al.^44^; Kuhl et al.^45^; Menell et al^46^

BCT, breast-conserving therapy; HER-2; Luminal B; HG, high-grade; DCIS, ductal carcinoma in situ; MRI, magnetic resonance imaging.

The breast cancer subgroups in which pre-treatment MRI has been shown to be particularly beneficial are:

- invasive lobular carcinoma^40,41^
- triple negative or basal carcinoma^42^
- luminal B and Her-2 carcinomas^43^
- intermediate and high-grade DCIS.^37,44^

Magnetic resonance imaging has been shown to be the most sensitive modality for demonstrating the presence and extent of DCIS.^6,45^ This, however, must be qualified. Magnetic resonance imaging is not sensitive for low-grade DCIS. It is, however, sensitive for medium- to high-grade DCIS and must be used appropriately.^44^ It is particularly useful in younger patients and patients with dense breast tissue. It will often show extent of disease far in excess of that represented on mammogram by micro-calcifications alone^46^ (see Table 3).

**TABLE 3:** Sensitivity and specificity of magnetic resonance imaging in diagnosing ductal carcinoma in situ.

Modality	Sensitivity (%)	Specificity (%)
MRI	98/91/80	81–98
Mammography	20–50	93–98

*Source*: Raza et al.^6^; Kuhl et al.^37^; Eun et al.^44^; Kuhl et al^45^

MRI, magnetic resonance imaging.

Breast MRI as part of a pre-treatment work-up is generally not considered appropriate in patients scheduled for mastectomy. However, an exception may be in the case where a nipple sparing mastectomy is being considered and MRI is used to rule out cancer invasion of the nipple–areola complex.^47^

**FIGURE 5:**
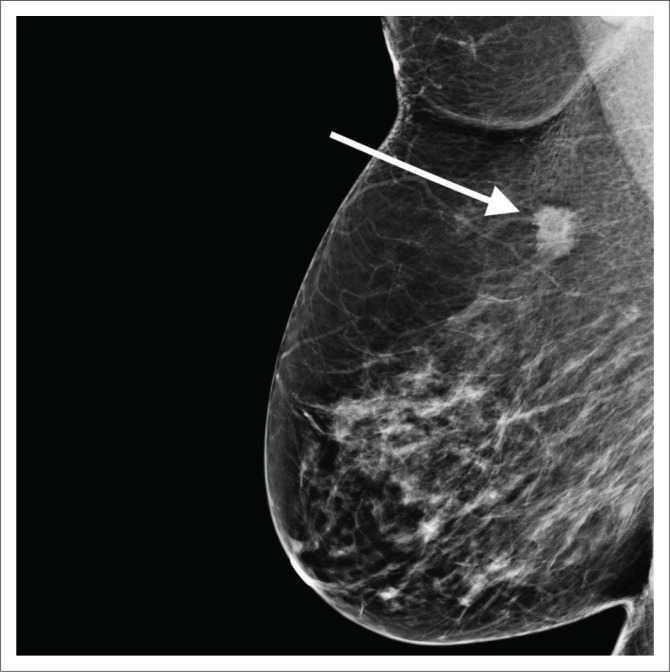
Mammogram (RMLO) in a 70-year-old patient shows a solitary spiculated mass in the upper breast (white arrow). There is clearly no invasion of surrounding tissue and absence of dense fibroglandular tissue that may be obscuring multifocal or multicentric disease. Biopsy revealed a low-grade luminal A cancer. This patient was not a candidate for pre-operative breast magnetic resonance imaging. RMLO, Right medio-lateral Oblique view.

**FIGURE 6:**
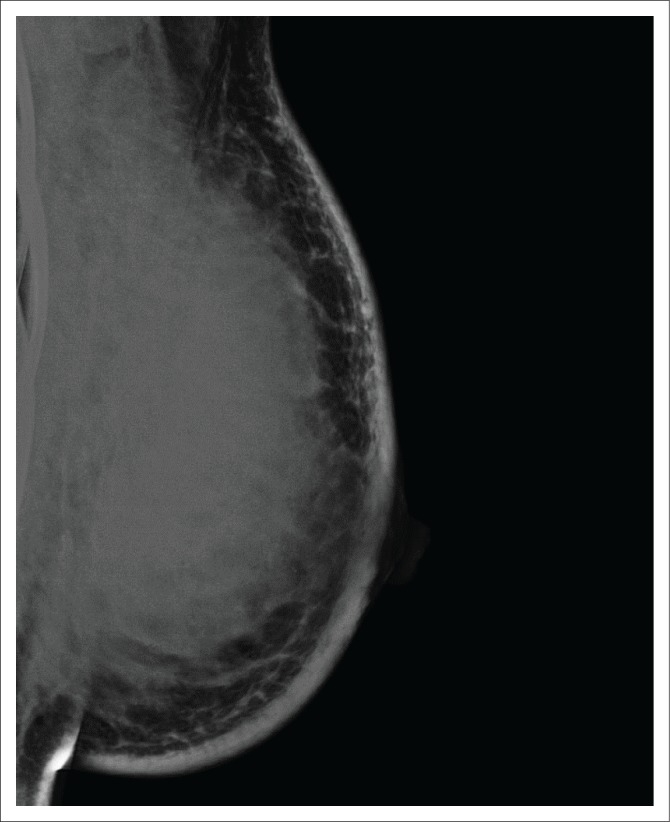
Mammogram (Left Medio-Lateral Oblique view) in a 39-year-old patient with a mass that fills the entire breast. There are inflammatory changes and extensive lymph node metastases. This patient was not a candidate for pre-treatment breast magnetic resonance imaging.

Patients with low grade, hormone-responsive solitary cancers in fatty breasts are less likely to benefit from pre-treatment breast MRI (see Figure 5). Likewise locally advanced cancers with clear involvement of skin and/or nipple areola complex, usually do not warrant MRI staging (see Figure 6).

Lastly, modern MRI staging is intimately tied to MRI-guided biopsy and localisation. The original research relied upon MRI findings not backed up by tissue diagnosis. Consequently, positive MRI findings necessitated upstaging and more extensive surgery. Hence, the higher mastectomy rate without necessarily improving clinical outcomes. Magnetic-resonance-imaging-guided biopsy allows us to confirm the presence of actual malignancy. Similarly, MRI-guided localisation means accurate resection of affected areas identified on pre-operative MRI.^12,48,49,50^

## Equivocal findings on mammogram and ultrasound – Problem solving

Magnetic resonance imaging must establish presence or absence of disease, not likelihood of malignancy.^51,52^ An attempt to standardise the role of MRI as a relevant problem solver means having guidelines based on certain mammogram features. The best proposal so far is that any abnormal finding that may represent a subtle cancer, but is not amenable to ultrasound or stereotactic biopsy, should be further evaluated on MRI.^53,54^ A suspicious area on mammogram (BI-RADS 4) that is not identifiable on ultrasound and not accessible for stereotactic biopsy (tomosynthesis-guided biopsy may mean that even single-view abnormalities are often amenable to biopsy) should be assessed on MRI. An example is a new or larger asymmetry or area of architectural distortion seen only on one mammographic view, which is not ultrasound visible and cannot be accessed with guided biopsy. When such an abnormality is seen at baseline mammogram, it should be considered as BI-RADS 3 that can be reviewed on mammogram in 6 months^55^ (see Table 4).

**TABLE 4:** Appropriateness of breast magnetic resonance imaging in indeterminate mammogram or ultrasound cases.

Appropriate	Inappropriate
Single-view asymmetries and/or architectural distortion	Masses on mammography and/or sonar
Discordant findings	Focal asymmetries – two views
Extremely dense, complex breasts on mammography and sonar	Suspicious micro-calcifications
-	Breast magnetic resonance imaging should not replace biopsy

*Source*: Giess et al.^51^; Ozcan et al.^52^; Moy et al.^53^; Bennani-Baiti et al.^54^; Bowles et al^55^

**FIGURE 7:**
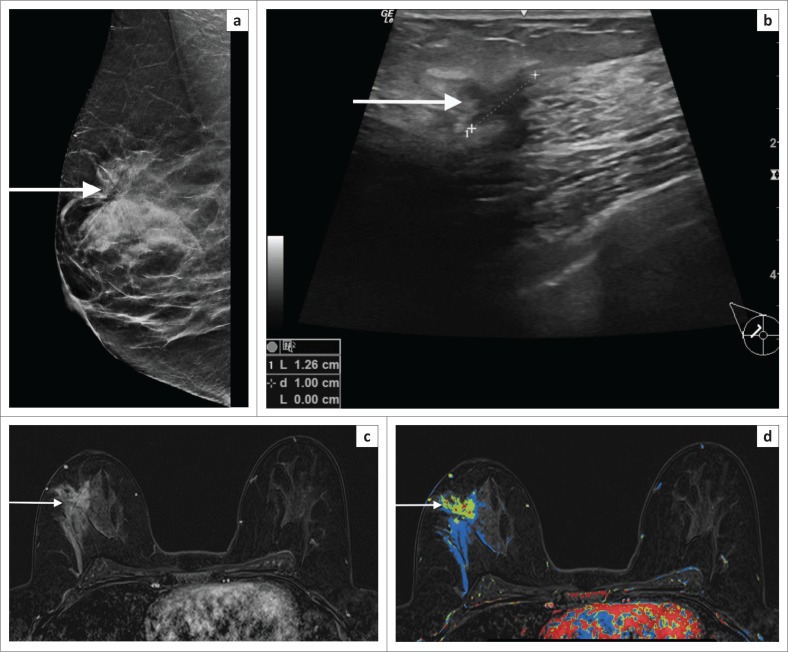
(a) Mammogram (LCC) in a 42-year-old patient who had an area of architectural distortion in the upper half of the right breast (white arrow). (b) Ultrasound indicated a suspicious mass (white arrow) – irregular, anechoic mass with an echogenic halo. However, histology indicated a complex sclerosing lesion. This was felt to be discordant. (c) Post-contrast magnetic resonance imaging shows a spiculated solid mass (white arrow). (d) The colour overlay shows washout and plateau features. Repeat biopsy-confirmed invasive carcinoma.

Any BI-RADS 4 area that can be biopsied under either ultrasound or mammography guidance (stereotactic or tomosynthesis) must be biopsied. Magnetic resonance imaging should not be used in these circumstances. Magnetic resonance imaging must never replace biopsy. Magnetic resonance imaging for equivocal mammogram or ultrasound findings needs to be used judiciously in order to avoid overuse^52^ (see Figure 7).

## Post-surgical and post-treatment magnetic resonance imaging

Post-treatment MRI indications fall into the following categories:

- Early post-surgery to identify residual disease or need for re-excision.^56,57^
- Differentiating cancer recurrence from scarring or fat necrosis.^58,59^

We still see patients who have undergone surgical excision as the primary histological diagnostic procedure. Much of the time, there remains positive cancer margins.^56^ There appears to be a correlation between volume of residual disease and MRI sensitivity. Below 5 mm, a residual focus is difficult to distinguish from benign post-surgical change.^57^ In order to determine the extent of residual disease and to aid decision-making with regard to re-excision or mastectomy, it is often valuable to perform an MRI.

A similar situation sometimes occurs with a vacuum-assisted biopsy of a small mass or small cluster of micro-calcifications (DCIS). Vacuum biopsies often remove the entire cancer but in many cases residual or synchronous disease remains.

Although post-surgical changes including haematoma and enhancing granulation tissue can make diagnosis of residual cancer tricky, there is still a role for MRI in the post-surgical setting. Magnetic resonance imaging performed in the early post-surgical period – up to 1 week post-procedure – usually avoids the misleading post-surgical enhancement that develops slightly later.^56^

**FIGURE 8:**
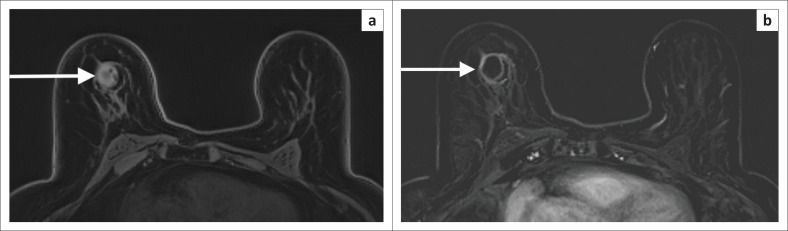
A 48-year-old woman who recently underwent a vacuum-assisted biopsy of a small mass in the right breast. Histology confirmed an invasive ductal carcinoma. Residual carcinoma was suspected. (a) Pre-contrast T1 fat-saturated MRI shows a hyperintense round mass with irregular margins (white arrow). The hyperintense signal is because of haematoma. (b) Post-contrast-subtracted image shows rim-enhancing mass (white arrow) in the right breast. The rim is thin and uniform. No thickened areas or mural nodules to suggest residual disease.

In more delayed scans, it is important to differentiate fat necrosis, scarring or seroma (with an enhancing wall) from residual cancer (see Figure 8). Residual cancer at the edge of collections and cysts has several characteristic features such as thick irregular wall enhancement and nodular enhancement.^58,59^

**FIGURE 9:**
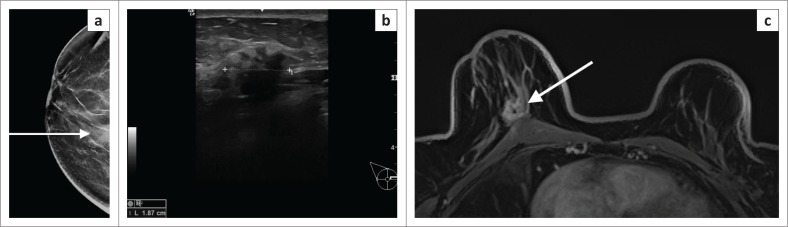
A 43-year-old patient treated 2 years earlier for high-grade invasive ductal carcinoma in the right breast now has a palpable lump along the right chest wall. (a) Mammogram shows a focal asymmetry which is otherwise difficult to characterise because of the posterior position. (b) Ultrasound shows a mixed echogenicity mass which appears to contain fat. This is suggestive of fat necrosis. (c) Post-contrast MRI shows an irregular, spiculated mass against the pectoral muscle. Biopsy was performed. High-grade breast cancer recurrence was confirmed at histology.

Breast cancer recurrence in an altered (post-surgery and radiation) breast can be difficult to identify clinically, on mammogram and ultrasound because of the overlap between scarring, fat necrosis and radiation related inflammatory change. MRI is often more accurate at demonstrating malignant from benign post treatment changes^59^ (see Figure 9).

Most post-treatment lesions can be diagnosed on mammogram and/or ultrasound. The decision as to whether MRI should be performed to resolve the difference between fat necrosis or scarring and recurrent cancer is based on the same principles used when using MRI for equivocal findings.^53^ If a suspicious area is identified on mammogram or ultrasound and is amenable to biopsy, MRI is not indicated. If, however, there is a discordant biopsy result or the area is not amenable to biopsy up front, MRI should be considered.

## Implant assessment

**FIGURE 10:**
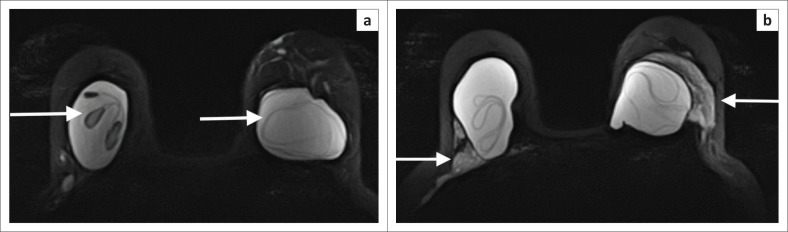
Non-contrast, polarity-altered spectral and spatial selective acquisition (PASTA) sequences to highlight silicone prostheses. (a) The linguine sign (white arrows) in both silicone prostheses. This indicates intra-capsular rupture. (b) In addition to the intra-capsular rupture, there are areas of high signal outside the implants (white arrows) in keeping with extra-capsular rupture and silicone extravasation.

Magnetic resonance imaging is the most accurate test to assess integrity of implants.^60^ Silicone specific,T1 and T2 sequences clearly demonstrate intra and extracapsular rupture (see Figure 10). Nonetheless, clinical guidance is necessary to determine the benefit of MRI. If a patient is symptomatic and/or surgery for a damaged prosthesis is being considered, MRI may be useful to assist in treatment planning.

Breast MRI is more sensitive for picking up cancers in women with prostheses than mammogram or ultrasound.^61^

Although excellent in demonstrating fluid around prostheses, it cannot reliably identify large cell lymphomas.^60^

### Treatment monitoring

Magnetic resonance imaging should be considered whenever a patient is scheduled for Neoadjuvant Chemotherapy (NAC). Although ultrasound is generally accurate for assessing change in size (and derived volume) of a tumour mass, MRI is now considered the most accurate imaging method to assess response to NAC.^62^

This was similarly demonstrated in the American College of Radiology Imaging Network (ACRIN) 6657 I-Spy 1 trial,^63^ a prospective, multi-institutional trial that validated the accuracy of breast MR imaging for assessment of neoadjuvant therapy response.

Some cancers may be non-viable or replaced by fibrosis post chemotherapy, but a residual mass remains. Magnetic resonance imaging, by way of its functional capabilities, can reflect tumour viability. This relates to the neovascularity and vascular permeability of viable cancers – indicated by contrast enhancement reflecting vascular changes in the cancer.

In addition, functional MRI protocols such as DWI can indicate non-viable tumour because of loss of restriction of molecules within the cancer post-chemotherapy.^62^ The ACRIN 6698 I-Spy 2 trial is assessing the utility of DWI and chemotherapy response.^64^

Magnetic resonance imaging is particularly accurate at monitoring response of Her-2 and triple negative cancers being treated with NAC.^65^ It is recognised that NAC that includes a Taxane may result in underestimation of residual disease because Taxanes reduce perfusion independent of the cytotoxic effect these drugs exert. Nonetheless, there is great value in MRI assessment of response to NAC as early non-responders can be identified at an early stage. At this stage, MRI is more useful to assess early response rather than establish complete post-treatment resolution.^12,66^

The guideline for MRI monitoring of chemotherapy is to perform three scans: the first scan is performed prior to chemotherapy initiation, the second halfway through the chemotherapy regimen and the third is performed at the end of treatment. Comparison of tumour volume and perfusion and/or diffusion characteristics should be made on each scan.^12^

### Magnetic resonance image-guided biopsy

It is an accepted principle of image-guided biopsies that a lesion visible only on a particular modality should be amenable to biopsy guided by that modality. In order to truly capitalise on the high sensitivity of MRI – which identifies otherwise occult primary cancers and determines true disease extent and multifocal or multicentric cancer – it is imperative that any finding that may affect treatment is confirmed histologically or demarcated by wire localisation.^67^

Second look (targeted) ultrasound following the discovery of additional disease on breast MRI is strongly advised and an ultrasound-guided biopsy should be performed if the lesion is visible. However, less than half of MRI-detected lesions are visible on targeted ultrasound.^68^

**FIGURE 11:**
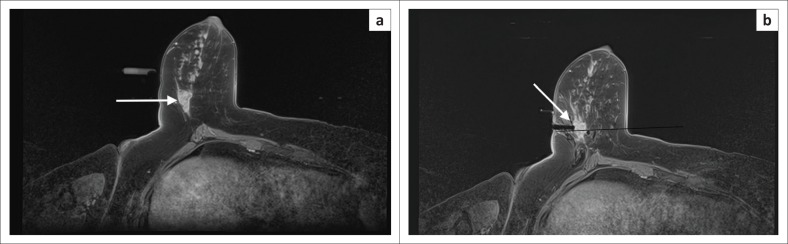
Magnetic-resonance-imaging-guided biopsy. (a) A 44-year-old patient with segmental non-mass enhancement (white arrow) in the right axillary tail. Not adequately seen on mammogram. (b) Vacuum-assisted biopsy ready. Obturator in place – tip corresponds to centre of needle trough (white arrow). Histology confirmed high-grade DCIS.

Any centre offering breast MRI, whether for high-risk screening, pre-operative staging or any of the other indications, should be able to perform MRI-guided biopsy. Alternatively, an arrangement with a department that does offer MRI-guided biopsies should be in place^37,67^ (see Figure 11).

Magnetic resonance image-guided biopsy wire localisation means that an affected area (identified on MRI) can be accurately marked out for the surgeon.

Magnetic resonance image-guided biopsy biopsies are performed using a specialised grid that is digitally superimposed on the breast of interest. A contrast-enhanced, fat-saturated scan is performed and the location of the suspected cancer or DCIS is determined as a grid coordinate. Together with calculated depth, this allows for accurate placement of a stylet. Vacuum-assisted core biopsies are usually performed and tissue markers always inserted post-procedure. Wire localisation is performed in a similar way, substituting a wire for the biopsy device.

## Discussion

Breast MRI used as a screening test in women at high risk of breast cancer has been shown to be significantly superior to mammography and ultrasound. At present, it is underutilised.^69,70^ This is largely because of clinicians not being aware of the benefits that MRI offers to patients in a high-risk category.^19^

Screening for high-risk patients applies in the South African context as much as the rest of the world. Most medical aids do pay for screening MRI in high-risk patients and Discovery Medical Scheme, in particular, is emphasising breast MRI in eligible patients.^71^ Although there is obviously limited access to MRI facilities in most of the public hospital sector, the policy document on breast cancer control put out by the Department of Health also advocates breast MRI in high-risk patients.^72^

As with many complex imaging scenarios, there is much to consider when looking at pre-operative scanning. The ability to detect additional multifocal and/or multicentric disease, as well as intra-ductal components allows for more accurate surgical planning and fewer positive margins post-surgery. This advantage is largely dependent on the ability to biopsy additional disease by way of targeted post-MRI ultrasound or under MRI guidance. Similarly, MRI-guided localisation defines information about disease extent that can be transferred from the MRI scanner to the theatre.^37^

Some centres have the policy that there is benefit from performing pre-operative MRI in all breast cancer patients undergoing conservative surgery.^73^ There remains debate about the role of pre-operative breast MRI, although the tide appears to be shifting in favour of MRI, especially in higher risk patients and higher grade cancers.^73^ Strong consideration for pre-treatment MRI should be given to certain sub-populations of breast cancer such as invasive lobular, HER-2, triple negative, high-grade DCIS and cancers in young women or high-risk groups.^40,42,43^ Although mastectomy is generally regarded as a contraindication to pre-operative breast MRI, in the setting of a planned skin- or nipple-sparing mastectomy, pre-operative MRI is useful for excluding any cancer invasion or proximity to skin or nipple–areola complex.^47^

It has also become clear that women with a personal history of breast cancer, especially those who have undergone breast-conserving therapy or are otherwise considered as high risk (BRCA, family history, dense breast tissue), should have access to MRI breast screening.^19,28^

As noted above, there is no consensus in South Africa about the role in pre-operative MRI. What is advisable though is discussion of the pros and cons in each case within the setting of a multidisciplinary meeting.^74^ With appropriate consultation and motivation from treating clinicians, most medical aids are willing to authorise pre-operative MRI staging.

Breast MRI is an excellent problem solver because of its high sensitivity and NPV.^51^ As breast imagers, everyone encounters difficult mammogram and ultrasound patients. With access to an MRI facility, it is always tempting to obtain answers with an MRI scan. However, it is neither practical nor beneficial to go down this road without due caution. There are most certainly situations where indeterminate cases need the tools that MRI can provide but it should only be considered when ultrasound or stereotactic biopsy is not practical.

Occasionally, high breast density compounded by the presence of scarring, fibrocystic disease, free silicone or other obscuring structures makes mammogram and ultrasound extremely difficult to interpret. Magnetic resonance imaging may be the only way to reliably rule out cancer in these situations.

In terms of MRI for monitoring of neoadjuvant therapy, it is becoming clearer that MRI has advantages over ultrasound.^65^ A significant percentage of cancers are not well visualised on ultrasound. Magnetic resonance imaging, as we know, is far more sensitive that mammogram or ultrasound for showing extent and multifocality of breast cancers. Magnetic resonance imaging is particularly helpful early on in treatment to show non-responders. However, it can also be used to show the absence of disease at the end of chemotherapy. In this respect, it has a high NPV although the positive predictive value is less impressive. (A negative post-treatment MRI is highly reliable in demonstrating pathological complete response).^65,66^ Once again, an individualised approach, taking into account specific cancer and patient factors, is advised.

In South Africa, breast MRI is only available to a small portion of the population, those who have access to private health facilities or academic hospitals. Within that environment, we must make sure that we use it appropriately and judiciously.

Breast MRI is a costly examination that is one of the main reasons limiting its widespread usage and opposition from some quarters. An abbreviated breast MRI protocol has been shown to be almost identical in terms of accuracy to a full length scan.^75^ There is a possibility in the near future that MRIs of the breast can even be carried out without contrast, using radiomics and DWI kurtosis techniques.^76^ Already quantitative apparent diffusion coefficient (ADC) evaluation is showing great promise in distinguishing benign from malignant tissue.^15^ These advances will hopefully help reduce costs and make clinical indications the most important determinant of breast MRI application, rather than simply cost.

## Implications and recommendations

- Annual breast MRI is recommended for high-risk patients, most particularly those who have a higher than 20% lifetime risk of developing breast cancer. It is imperative that this message is distributed not only by radiologists, but also by breast surgeons, gynaecologists and genetic counsellors.
- Breast MRI in the setting of problem solving, pre-operative staging and investigating residual disease or recurrence needs to be decided upon in the setting of a multidisciplinary team. Evidence-based practice, consistent methodology and patient benefit need to be taken into consideration with each case.
- Breast MRI should be performed at units capable of doing MRI-guided biopsy and localisation. Alternatively, breast MRI units should have arrangements with other departments that have the interventional capacity.
